# Risk Perceptions for Avian Influenza Virus Infection among Poultry Workers, China 

**DOI:** 10.3201/eid1902.120251

**Published:** 2013-02

**Authors:** Qi Yu, Linqing Liu, Juan Pu, Jingyi Zhao, Yipeng Sun, Guangnian Shen, Haitao Wei, Junjie Zhu, Ruifeng Zheng, Dongyan Xiong, Xiaodong Liu, Jinhua Liu

**Affiliations:** Author affiliations: Beijing General Station of Animal Husbandry and Veterinary Medicine, Beijing, People’s Republic of China (Q. Yu, J. Zhao, H. Wei, J. Zhu, X. Liu);; China Agricultural University, Beijing, People’s Republic of China (J. Liu, L. Liu, J. Pu, Y. Sun);; Beijing Laboratory Diagnosis Institute of Veterinary Medicine, Beijing, (G. Shen, R. Zheng);; Pinggu Animal Disease Control and Prevention, Beijing (D. Xiong);; Shandong Animal Disease Control Center, Jinan, China (J. Liu)

**Keywords:** Avian influenza, influenza, viruses, poultry workers, serologic survey, knowledge, attitudes, practices

## Abstract

To determine risk for avian influenza virus infection, we conducted serologic surveillance for H5 and H9 subtypes among poultry workers in Beijing, China, 2009–2010, and assessed workers’ understanding of avian influenza. We found that poultry workers had considerable risk for infection with H9 subtypes. Increasing their knowledge could prevent future infections.

Avian influenza A viruses (AIVs), subtypes H5N1 and H9N2, are endemic to poultry in the People’s Republic of China and have often infected humans. During early 2009, several cases of subtype H5N1 infection were found in China (*1*), and on January 6, a case was confirmed in a girl in Beijing. Clinical data showed that the girl had contact with slaughtered ducks, which were bought from a farm product market in Yanjiao, Langfang, Hebei Province, which neighbors Beijing. To assess the risk for AIV infection among poultry workers, we conducted serologic surveillance in Beijing from May 2009 to March 2010. Using a questionnaire, we also assessed the knowledge, attitudes, and practices (KAPs) of poultry workers regarding avian influenza infection. The Ethics Committee of Beijing Municipal Bureau of Agriculture approved this study, and all participants signed informed consent documents.

## The Study

A total of 305 serum specimens were collected from 305 workers who were in close contact with poultry populations during May 2009–March 2010. Influenza strains A/duck/Huabei/01/2007 (H5N1), belonging to clade 2.3.4, and A/chicken/Shangdong/ZB/2007 (H9N2) of the F/98 genotype were used for the microneutralization assay, which was performed as described (*2*,*3*). The F/98 genotype (H9N2) and clade 2.3.4 (H5N1) viruses had been demonstrated to be the predominant strains circulating in poultry in this region and were responsible for most cases of human infection during the period of the survey (*4**,**5*). Therefore, we only used the 2 viral strains in the MN assay. 

Serum samples were considered positive if titers were >80, and all results were generated from at least 2 independent assays. Simultaneously, the 305 surveyed workers were administered questionnaires to ascertain avian influenza–related KAPs. Among the distributed questionnaires, responses from 207 were considered valid and were used for further analysis. Epi Info software, version 3.5.4 (Centers for Disease Control and Prevention, Atlanta, GA, USA), was used to analyze the survey data. The Pearson χ^2^ test was used to compare differences between groups. Differences were considered significant if p value was <0.05.

Of the 305 poultry workers, 155 (50.8%) were duck keepers from 8 farms, 114 (37.4%) were chicken keepers from 5 farms, and 36 (11.8%) were chicken butchers who worked at an abattoir. The duck and chicken farms were located in different districts in Beijing. One hundred and fifty-five (50.8%) workers were male, and 150 (49.2%) were female; 147 (48.2%) participants were 36–45 years of age, 76 (24.9%) were 18–35 years, and 82 (26.9%) were >45 years. All participants had no history of vaccination for seasonal influenza in the past 3 years. MN assay revealed that no workers were positive for antibodies against influenza virus subtype H5, whereas 14 (4.6%) were positive for antibodies against subtype H9; titers ranged from 80 to 640 (Figure). Further analysis indicated that proportions of seropositive workers were 2.6% (4/155) for men and 6.7% (10/150) for women. By age, the proportions of seropositive poultry workers were 9.2% (7/76) for those 18–35 years, 2.7% (4/147) for those 36–45-years, and 3.7% (3/82) for those >45 years of age. These results suggest that subtype H9N2 virus infection was more prevalent among persons 18–35 years of age. The proportions of seropositive duck keepers, chicken keepers, and chicken butchers were 3.9% (6/155), 3.5% (4/114), and 11.1% (4/36), respectively. No significant differences were found in the infection rate among the 3 groups.

**Figure:**
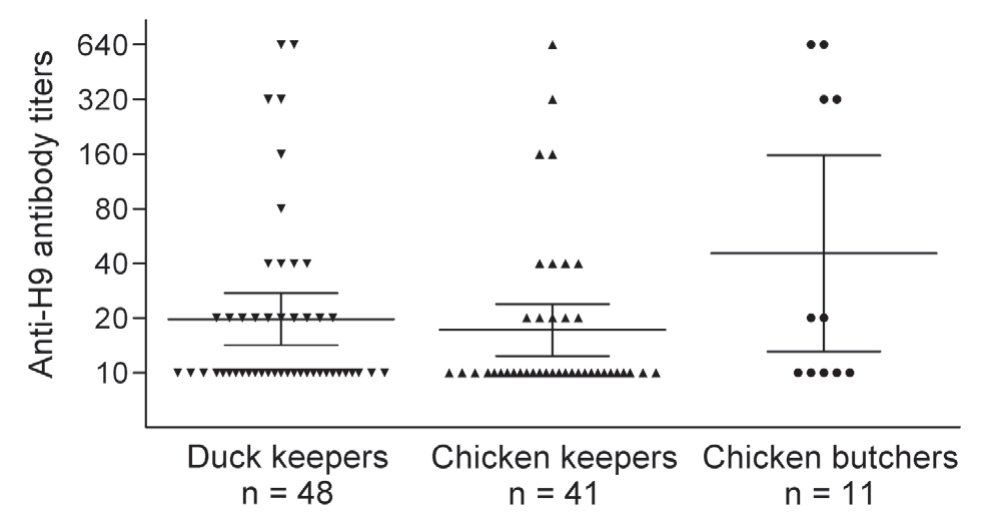
Avian influenza A (H9N2) virus microneutralization titers of workers with occupational exposure to poultry, Beijing, China, 2009–2010. A total of 305 serum specimens were tested by microneutralization assay, serum samples were considered positive with titers >80, and titers <10 were not included in this figure. Geometric mean titers and 95% CIs of subtype H9N2 microneutralization titers in various groups are indicated by long and short horizontal lines.

A total of 207 poultry workers completed a questionnaire regarding KAPs related to avian influenza. In terms of knowledge of avian influenza, 181 (87.4%) of workers recognized the transmission route through the respiratory tract, 113 (54.6%) recognized the transmission route through the gastrointestinal tract, and 117 (56.5%) recognized the transmission route through the mucosa. Nearly half of the participants ignored the latter 2 major transmission routes. In terms of knowledge of poultry housing practices, 135 (65.2%) and 160 (77.3%) of the workers had the correct understanding that chickens and ducks should not be raised with pigs in the same backyards and that poultry should not have contact with wild birds. Furthermore, 181 (87.4%) of the participants knew that eating and selling dead birds was against health regulations, and 167 (80.7%) knew that improving vaccination coverage and quality is an effective strategy for preventing AIV infection. Significant discrepancies were observed among groups with different educational levels in knowledge of avian influenza (except for those who understood that infection occurred through the respiratory tract and mucosa) (p<0.05) (Table 1). Workers with a high level of education (senior high school, university or college, and above) had more correct answers to the corresponding questions. Significant differences were also found between groups of different ages and occupations regarding knowledge of avoiding mixed housing practices (p<0.05). Most (79.0%–95.0%) young persons, 18–35 years of age, knew that poultry should not be kept in mixed housing with pigs nor kept in contact with other species of birds. Also concerning the above 2 risks, chicken keepers had more accurate knowledge than duck keepers (Table 1). No significant differences were found between men and women in terms of general knowledge (p>0.05). 

**Table 1:** Knowledge of avian influenza among 207 poultry workers, Beijing, China, 2009–2010*

Risk variable†	OR‡	95% CI	p value
AIV infection through the respiratory tract			
Age, y (<36/36–45/>45)	–/1.97/1.11	–/0.66–6.11/0.27–4.46	–/0.18/0.87
Education (low/high)	–/0.57	–/0.21–1.53	–/0.23
Job (chicken keepers/duck keepers)	–/1.11	–/0.44–2.79	–/0.81
AIV infection through the gastrointestinal tract			
Age (<36/36–45/>45)	–/1.03/0.94	–/0.52–2.04/0.42–2.11	–/0.93/0.88
Education (low/high)	–/0.49	–/0.26–0.91	–/**0.02**
Job (chicken feeders/duck feeders)	–/1.52	–/0.83–2.78	–/0.14
AIV infection through mucosa			
Age (<36/36–45/>45)	–/0.85/0.64	–/0.43–1.68/0.28–1.44	–/ 0.61/0.24
Education (low/ high)	–/0.51	–/0.27–0.95	–/**0.02**
Job (chicken feeders/duck feeders)	–/1.58	–/0.86–2.89	–/0.11
Avoiding mixed housing with pigs			
Age, y (<36/36–45/>45)	–/2.54/2.69	–/1.17–5.6/1.11–6.6	–/**0.01**/**0.02**
Education (low/ high)	–/0.21	–/0.10–0.44	–/**<0.01**
Job (chicken feeders/duck feeders)	–/3.97	–/1.96–8.14	–/**<0.01**
Avoiding touching wild birds			
Age (<36/36–45/>45)	–/7.95/12.87	–/2.14–34.91/3.24–59.52	–/**<0.01**/**<0.01**
Education (low/high)	–/0.00	–/0.00–0.11	–/**<0.01**
Job (chicken feeders/duck feeders)	–/–	–/–	–/ **<0.01**
Forbidding eating and selling dead birds			
Age, y (<36/36–45/>45)	–/1.1/2.82	–/0.33–3.70/0.87–9.44	–/0.87/**0.05**
Education (low/high)	–/0.05	–/0.01–0.22	–/**<0.01**
Job (chicken keepers/duck keepers)	–/1.63	–/0.63–4.33	–/0.28
Improving vaccination coverage and quality			
Age, y (<36/36–45/>45)	–/0.79/0.93	–/0.33–1.90/0.34–2.52	–/0.57/0.87
Education (low/high)‡	–/0.14	–/0.04–0.44	–/**<0.01**
Job (chicken keepers/duck keepers)	–/1.03	–/ 0.48–2.21	–/0.93

*OR, odds ratio; AIV, avian influenza virus; –, OR of variable itself is not calculated; **boldface** indicates that p value is significant. †Low education indicates junior high school, elementary school, and below; high education indicates senior high school, university or college, and above. ‡ORs are calculated as follows: for different age groups, we calculated 2 ORs—OR1 = odds (<36 y)/odds (36–45 y), OR2 = odds (<36 y)/odds (>45 y); for different education groups, OR = odds (low)/odds (high); for different job groups, OR = odds (chicken keepers)/odds (duck keepers).

Regarding attitudes toward avian influenza, 116 (56.0%) of 207 surveyed workers did not consider that AIVs pose a public health threat. They also rarely showed concern for the consequences resulting from avian influenza. 

Analysis of practices concerning avian influenza prevention among 207 poultry workers is shown in Table 2. Although 184 (88.9%) respondents said they wore specific work clothing, wearing personal protective equipment was not a routine practice among poultry workers: only 112 (54.1%) wore gloves, and 95 (45.9%) wore masks. We also found that 165 (79.7%) participants routinely washed their hands after work and that 174 (84.1%) workers regularly used disinfectant. Significant differences were found between chicken keepers and duck keepers; the former were more likely to follow good hygiene practices than were the latter (p<0.01) (Table 2). 

**Table 2:** Practices of avian influenza among 207 poultry workers, Beijing, China 2009–2010*

Risk variable†	OR‡	95% CI	p value
Wearing work clothing			
Age, y (<36/36–45/>45)	–/3.08/11.24	–/0.58–21.83/2.22–76.60	–/0.19/**<0.01**
Education (low/high)	–/0.70	–/0.25–1.91	–/0.45
Job (chicken keepers/duck keepers)	–/0.2	–/0.07–0.58	–/**<0.01**
Wearing gloves			
Age, y (<36/36–45/>45)	–/0.51/0.62	–/0.26–1.02/0.27–1.38	–/**0.04**/0.20
Education (low/high)	–/0.67	–/0.36–1.23	–/0.17
Job (chicken keepers/duck keepers)	–/0.43	–/0.23–0.78	–/**<0.01**
Wearing mask			
Age, y (<36/36–45/>45)	–/0.66/0.7	–/0.33–1.33/0.31–1.58	–/0.21/0.35
Education (low/high)	–/0.71	–/0.39–1.29	–/0.23
Job (chicken keepers/duck keepers)	–/0.42	–/0.23–0.77	–/**<0.01**
Washing hands after finishing work			
Age, y (<36/36–45/>45)	–/0.91/2.58	–/0.35–2.34/0.99–6.76	–/0.83/0.31
Education (low/high)	–/0.69	–/0.31–1.50	–/0.31
Job (chicken keepers/duck keepers)	–/0.29	–/0.14–0.63	–/**<0.01**
Regular disinfection			
Age, y (<36/36–45/>45)	–/1.52/4.29	–/0.49–4.86/1.39–13.77	–/0.43/**<0.01**
Education (low/high)	–/0.48	–/0.19–1.19	–/0.08
Job (chicken keepers/duck keepers)	–/0.11	–/0.04–0.30	–/**<0.01**


*OR, odds ratio; –, indicates that OR of variable itself is not calculated; b**oldface** indicates that p value is significant.  †Low education indicates junior high school, elementary school and below; high education indicates senior high school, university or college and above. ‡ORs are calculated as follows: for different age groups, we calculated 2 ORs—OR1 = odds (<36 y)/odds (36–45 y), OR2 = odds (<36 y)/odds (>45 y); for different education groups, OR = odds (low)/odds (high); for different job groups, OR = odds (chicken keepers)/odds (duck keepers).

## Conclusions

Transmission of AIVs from poultry to humans probably results from contact with infected poultry or contaminated materials (*6**–**9*). Workers in the poultry industry are at high risk for AIV infection. We found that 4.6% of poultry workers in Beijing had antibodies against influenza virus subtype H9. These findings indicate that viruses of subtype H9 may have previously infected a considerable number of persons in China, thus highlighting the potential public health risk for H9 AIV. None of the poultry workers in our study had positive test results for H5. Similarly, previous serologic surveillance studies in China showed that the prevalence of antibodies against H5 strains was significantly lower than that for antibodies against H9 (*10**,**11*). 
